# BMAL1 regulates mitochondrial homeostasis in renal ischaemia‐reperfusion injury by mediating the SIRT1/PGC‐1α axis

**DOI:** 10.1111/jcmm.17223

**Published:** 2022-02-17

**Authors:** Peng Ye, Wei Li, Xin Huang, Sheng Zhao, Wu Chen, Yuqi Xia, Weimin Yu, Ting Rao, Jinzhuo Ning, Xiangjun Zhou, Yuan Ruan, Fan Cheng

**Affiliations:** ^1^ Department of Urology Renmin Hospital of Wuhan University Wuhan China; ^2^ Department of Anesthesiology Renmin Hospital of Wuhan University Wuhan China

**Keywords:** BMAL1, mitochondrial biogenesis, renal IRI, SIRT1/PGC‐1α

## Abstract

The regulation of renal function by circadian gene BMAL1 has been recently recognized; however, the role and mechanism of BMAL1 in renal ischaemia‐reperfusion injury (IRI) are still unknown. The purpose of this study was to clarify the pathophysiological role of BMAL1 in renal IRI. We measured the levels of BMAL1 and mitochondrial biogenesis‐related proteins, including SIRT1, PGC‐1α, NRF1 and TFAM, in rats with renal IRI. In rats, the level of BMAL1 decreased significantly, resulting in inhibition of SIRT1 expression and mitochondrial biogenesis. In addition, under hypoxia and reoxygenation (H/R) stimulation, BMAL1 knockdown decreased the level of SIRT1 and exacerbated the degree of mitochondrial damage and apoptosis. Overexpression of BMAL1 alleviated H/R‐induced injury. Furthermore, application of the SIRT1 inhibitor EX527 not only reduced the activities of SIRT1 and PGC‐1α but also further aggravated mitochondrial dysfunction and partially reversed the protective effect of BMAL1 overexpression. Moreover, whether in vivo or in vitro, the application of SIRT1 agonist resveratrol rescued the mitochondrial dysfunction caused by H/R or IRI by activating mitochondrial biogenesis. These results indicate that BMAL1 is a key circadian gene that mediates mitochondrial homeostasis in renal IRI through the SIRT1/PGC‐1α axis, which provides a new direction for targeted therapy for renal IRI.

## 1 INTRODUCTION

Renal ischaemia‐reperfusion injury (IRI) is one of the main causes of acute kidney injury (AKI).^1^ Factors such as trauma, shock, cardiovascular surgery, sepsis and renal transplantation are the inducements of renal IRI. According to statistics, the incidence rate of AKI in hospitalized patients is between 2% and 7%, while in the intensive care unit, the incidence rate of AKI is >10%, with a mortality rate of 50%, which poses a great threat to the life and safety of patients.^2^ AKI caused by IRI often becomes an important risk factor for the progression of chronic kidney disease due to a series of complex pathophysiological processes, such as inflammation, apoptosis and fibrosis.^3^ Although there have been many studies on IRI, there is still a lack of effective treatment.^4^

In recent years, many studies have shown that the circadian clock plays an important regulatory role in maintaining the metabolism and homeostasis of many tissues, including the kidney.^5^ The circadian clock consists of a core component found in the hypothalamus suprachiasmatic nucleus and subordinate clocks located in peripheral tissues, and the circadian gene BMAL1 controls the positive and negative feedback loops of the central and peripheral clocks as a transcriptional activator. It regulates a wide range of physiological and metabolic processes through a transcriptional‐translational feedback loop that affects the expression of downstream target genes. Diseases such as hypertension, diabetes, cardiovascular disease and osteoarthritis have been found to be related to disruption of the BMAL1‐regulated circadian rhythm.^6^

Recent studies have indicated that BMAL1 may be involved in maintaining normal renal function.^7^ Knockout of BMAL1 in renal collecting duct and granular cells resulted in increased urine volume, decreased plasma aldosterone levels and decreased blood pressure.^8^ In whole nephron‐specific BMAL1‐knockout mice, the deletion of BMAL1 can lead to renal mitochondrial dysfunction and abnormal pharmacokinetics.^9^ In the model of renal fibrosis caused by unilateral ureteral obstruction, inducible BMAL1‐knockout mice whose gene deletion occurred in adulthood showed less renal tubular fibrosis.^10^ However, there is no evidence to clarify the role of BMAL1 in renal IRI.

Silent information regulator 1 (SIRT1) is a member of the class III HDAC family and is involved in regulating cell survival, metabolism, oxidative stress response, mitochondrial biogenesis and inflammation.^11^ SIRT1 also plays an important role in IRI. A decrease in its activity may lead to a decrease in ischaemia‐reperfusion (IR) tolerance in the kidney, myocardium, liver and brain.^12^, ^13^, ^14^ SIRT1 can also enhance mitochondrial biogenesis by activating peroxisome proliferator‐activated receptor gamma coactivator 1 alpha (PGC‐1α) and its downstream targets, such as nuclear respiratory factor 1 (NRF1) and mitochondrial transcription factor A (TFAM).^15^, ^16^ Furthermore, many studies have confirmed an interaction between SIRT1 and BMAL1. For example, SIRT1 alleviates injury by crosstalk with BMAL1 in a myocardial IR model.^17^ In cartilage, BMAL1 regulates the innate immune system through crosstalk with SIRT1,^18^ while in muscle and the liver, BMAL1 regulates insulin sensitivity by interacting with SIRT1.^19^, ^20^ However, the relationship between BMAL1 and SIRT1 in renal IRI is not clear. Therefore, we aimed to investigate the role of BMAL1 and SIRT1 in renal IRI and explore its molecular mechanism.

## 2 MATERIALS AND METHODS

### 2.1 Animals and experimental model of renal IRI

A total of 40 Sprague–Dawley rats aged 8–10 weeks and weighing 210–250 g were purchased from the Center of Experimental Animals at Wuhan University Medicine College (Hubei, China). All rats were housed in a standard temperature‐controlled room with an alternating 12 h light/dark cycle and provided ad libitum access to food and water. All experiments were approved by the Animal Experimental Ethics Committee of Wuhan University and complied with the Guide for the Care and Use of Laboratory Animals and AVMA Guidelines for the Euthanasia of Animals (Ethics Serial Number: WDRM 20200308A). Rats were randomly divided into four groups (*n* = 10): Sham (right nephrectomy), IR (ischaemia‐reperfusion), IR + Vehicle and IR + RES (resveratrol, HY‐16561, MedChemExpress). The rats in the IR + RES group were given resveratrol via gavage at a dose of 25 mg/kg/day for 2 weeks before the operation, while those in the IR + Vehicle group were given CMC‐Na. The renal IRI model was established as previously described.^21^ Rats were anaesthetized with 1% phenobarbital sodium at a dose of 50 mg/kg via intraperitoneal injection and kept warm with an electric blanket. A midline abdominal incision was made, the right kidney was removed by ligating the renal hilum, and then, the left renal hilum was ligated with a microvascular clamp. After 30 min, the clamp was removed to restore the left renal blood supply. The sham group only underwent a right nephrectomy without left hilum clamping. After 24 h, the rats were sacrificed with CO_2_ (30% of the chamber volume was displaced per minute with CO_2_), and the kidneys were carefully separated for follow‐up experiments.

### 2.2 Cell culture and establishment of a hypoxia and reoxygenation (H/R) model

HK‐2 cells were purchased from the Cell Bank of the Chinese Academy of Sciences and cultured in DMEM/F12 medium (SH30023.01B, HyClone) mixed with 10% foetal bovine serum (FBS) (SH30084.03, HyClone), 100 U/ml penicillin and 100 U/ml streptomycin in a 37°C humidified incubator containing 5% CO_2_, 21% O_2_ and 74% N_2_. The H/R model was established according to previously described methods.^22^ Briefly, the model was induced by replacing serum‐free DMEM/F12 pretreated in a three‐gas incubator for 24 h and placing the cells in a three‐gas incubator containing an atmosphere of 94% N_2_, 5% CO_2_ and 1% O_2_ for 24 h. After that, the serum‐free medium was replaced with complete medium, and the cells were restored to normoxic incubator conditions for 12 h before collection. The SIRT1 inhibitor EX527 (10 μM, HY‐15452, MedChemExpress) or agonist resveratrol (50 μM) was dissolved in DMSO and added to the medium 24 h before the H/R process.

### 2.3 Lentivirus infection and siRNA transfection

Methods for lentivirus infection and siRNA transfection can be found in the supplementary information.

### 2.4 Renal function measurement

Rat whole blood samples were collected and centrifuged at 500 g and 4°C for 15 minutes to obtain serum. According to the manufacturer's instructions, a creatinine assay kit (EICT‐100, BioAssay Systems), urea assay kit (DIUR‐100, BioAssay Systems) and automatic biochemistry analyzer (7600, Hitachi) were used to detect the levels of serum creatinine (Scr) and blood urea nitrogen (BUN) in the samples.

### 2.5 Antioxidant enzyme activity measurement

Methods for antioxidant enzyme activity measurement can be found in the supplementary information.

### 2.6 Haematoxylin and Eosin staining

Kidney tissue was washed with saline, fixed in a 4% paraformaldehyde solution, dehydrated using a gradient concentration of ethanol solutions, cleared in xylene, embedded in paraffin and cut into 4 µm sections. After dewaxing, hydration and haematoxylin/eosin staining, the degree of tissue injury was assessed by two independent pathologists using the Paller scoring system.^23^

### 2.7 TUNEL staining

An in situ cell death detection kit (11684817910, Roche) was used to evaluate the degree of renal apoptosis caused by IR, and all processes were completed according to the manufacturer's protocol. In short, paraffin sections were dewaxed and rehydrated using xylene and ethanol and immersed in protease K working solution for permeabilization. The samples were then incubated with 50 μl TUNEL reaction mixture at 37°C for 1 h. Finally, the samples were incubated with Converter‐POD, DAB substrate and haematoxylin in sequence, and the proportion of TUNEL‐positive cells in the samples was evaluated by two pathologists.

### 2.8 Immunohistochemical staining

Methods for immunohistochemical staining can be found in the supplementary information.

### 2.9 Immunofluorescence staining

Methods for immunofluorescence staining can be found in the supplementary information.

### 2.10 Transmission electron microscopy (TEM)

Fresh kidney tissue was fixed in 2.5% paraformaldehyde for 24 h and then fixed with 2% osmium tetroxide for 2 h. After dehydration with ethanol, the tissue was embedded in superepoxy resin and cut into 50 nm slices. Next, the slices were washed and stained with 2% aqueous uranyl acetate. Finally, the sections were visualized under a transmission electron microscope (H‐600, Hitachi).

### 2.11 Apoptosis assay

Methods for apoptosis assay can be found in the supplementary information.

### 2.12 Mitochondrial membrane potential (MMP) assay

Methods for MMP assay can be found in the supplementary information.

### 2.13 Western blot analysis

Treated cells or rat kidneys in a cold mixture of RIPA lysis buffer (P0013B, Beyotime) and PMSF (1 mM, ST505, Beyotime) were lysed with a cryogenic grinder or ultrasonic sonicator. After the lysate mixture was lysed on ice for 30 min, it was centrifuged at 8000 *g* for 15 min   at 4°C  to obtain supernatant. A Nuclear and Cytoplasmic Protein Extraction Kit (P0027, Beyotime) was used to extract cytoplasmic protein. Then, the protein concentration was measured using the BCA method on a microplate absorbance reader. Equal amounts of protein were separated via 12% SDS–PAGE, transferred onto polyvinylidene difluoride membranes (IPFL00010, Millipore) and then blocked with 5% nonfat milk for 1 h at room temperature. After that, the membranes were incubated with the following primary antibodies at 4°C overnight:anti‐BMAL1 (1:1000, NB100‐2288S, NOVUS), anti‐SIRT1 (1:1000, 8469S, CST),anti‐ PGC‐1α (1:1000, NBP1‐04676, NOVUS), anti‐TFAM (1:2000, 22586‐1‐AP, Proteintech), anti‐NRF1 (1:1000, 12482–1‐AP, Proteintech), anti‐Cytochrome *C* (Cyt *c*, 1:1000, ab13575, Abcam), anti‐caspase‐3 (1:1000, 19677‐1‐AP, Proteintech) and anti‐β‐actin (1:2000, GB11001, Servicebio). After three washes with TBST, the membranes were incubated with the corresponding HRP‐conjugated goat anti‐rabbit (1:10,000, BL003A, Biosharp) or goat anti‐mouse (1:10,000, BL001A, Biosharp) secondary antibody for 1 h. Finally, protein bands were visualized using a ChemiDoc imaging system (17001401, BIO‐RAD) with a supersensitive ECL substrate (BL520A, Biosharp) and analysed using ImageJ software (v 1.8.0, National Institutes of Health).

### 2.14 Gene expression and mtDNA analysis

Methods for gene expression and mtDNA analysis can be found in the supplementary information as well as the primer sequences.

### 2.15 Statistical analysis

All data are expressed as the mean ± standard deviation. GraphPad Prism (v9, GraphPad Software) was used to analyse data and construct graphs. Differences between groups were analysed via ANOVA followed by a post‐assay/test method involving a Bonferroni correction for a post hoc *t* test, and *p* < 0.05 was considered significant.

## 3 RESULTS

### 3.1 IR leads to aggravation of renal pathological damage, increased apoptosis and impaired renal function

**FIGURE 1:**
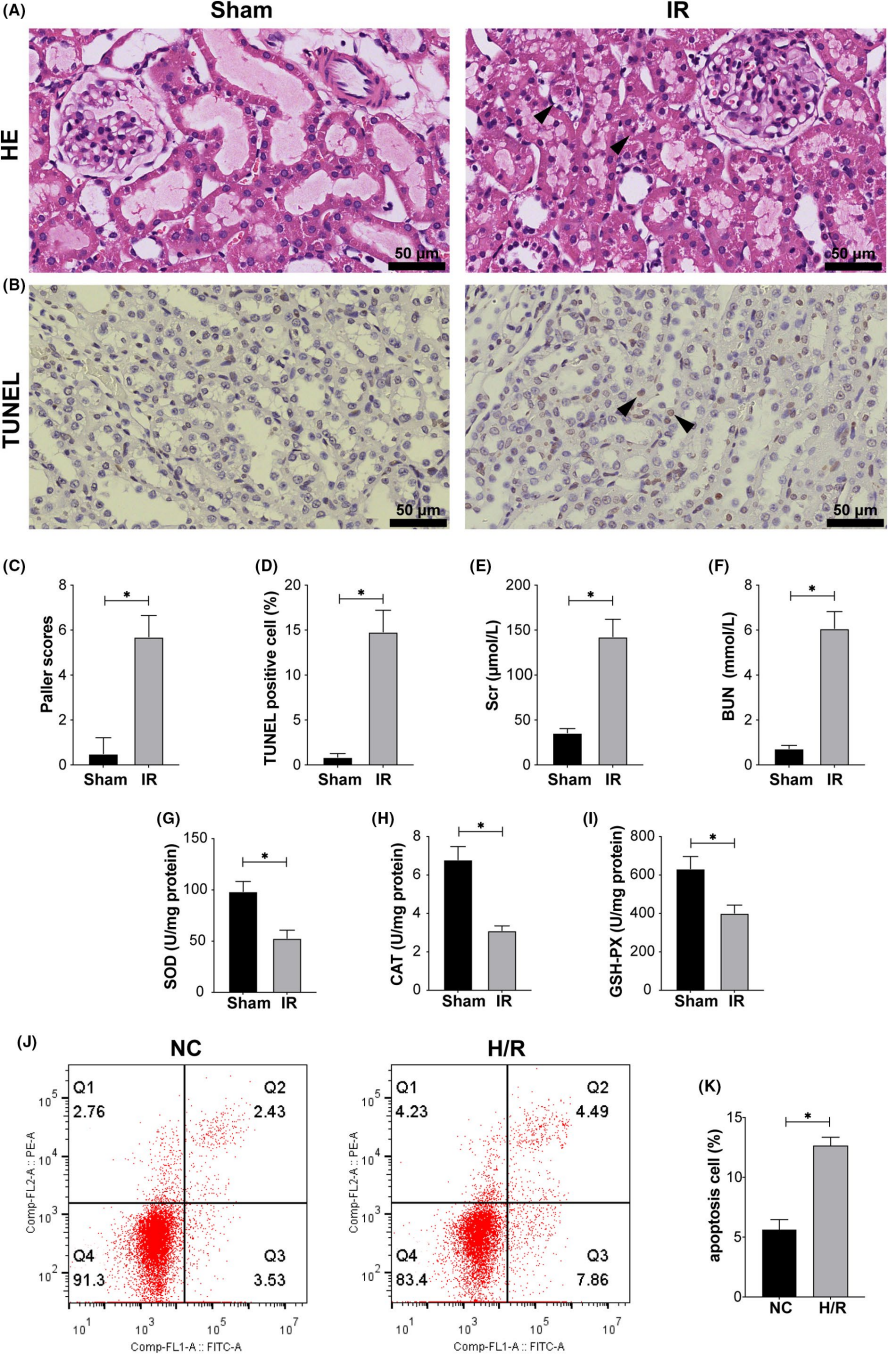
IR‐induced pathological injury and apoptosis in rat kidneys. (A) HE staining of rat kidneys after the IR procedure. The arrows in the figure indicate pathological damage (loss of brush border, haemorrhage, necrosis of renal tubular epithelial cells and inflammatory cell infiltration). Scale bar = 50 µm. Magnification ×400. (B) TUNEL staining was performed on rat kidneys. The arrows in the figure indicate examples of TUNEL‐positive cells. Scale bar = 50 µm. Magnification ×400. (C) Paller scores of the sham and IR groups after HE staining. (D) The proportion of TUNEL‐positive cells after TUNEL staining. (E) The content of Scr in the sham and IR groups. (F) The content of BUN in the sham and IR groups. (G) The activity of SOD in the sham and IR groups. (H) The activity of CAT in the sham and IR groups. (I) The activity of GSH‐PX in the sham and IR groups. (J, K) Flow cytometry analysis of the effect of H/R treatment on apoptosis and the quantitative values. Values are expressed as the mean ± SEM. ^*^ *p* < 0.05

After modelling, the renal tissue in the IR group showed more obvious histological damage than that in the sham group. H&E staining of sections showed that the renal tubular cells in the IR group were swollen, and extensive expansion and deformation of renal tubules, nuclear pyknosis and inflammatory cell infiltration were observed, while there were no obvious pathological changes in the sham group (Figure 1A). Furthermore, the Paller score in the IR group was also significantly higher than that in the sham group (Figure 1C). Meanwhile, TUNEL staining of sections showed that the proportion of apoptotic cells in the IR group increased significantly (Figure 1B,D). Scr and BUN levels also increased significantly after IR (Figure 1E,F). Similarly, IR significantly decreased the activities of SOD, CAT and GSH‐PX (Figure 1G–I). In vitro, HK‐2 cells were cultured in an H/R environment to simulate the process of IR in tissues. Flow cytometry showed that the apoptosis rate of HK‐2 cells cultured in the H/R environment increased significantly (Figure 1J,K). These results indicate that the degree of renal injury and apoptosis increased significantly after IR.

### 3.2 IR (H/R) decreased the expression of BMAL1 and downregulated SIRT1/PGC‐1α‐regulated mitochondrial biogenesis to exacerbate apoptosis in rat kidney/HK‐2 cells

**FIGURE 2:**
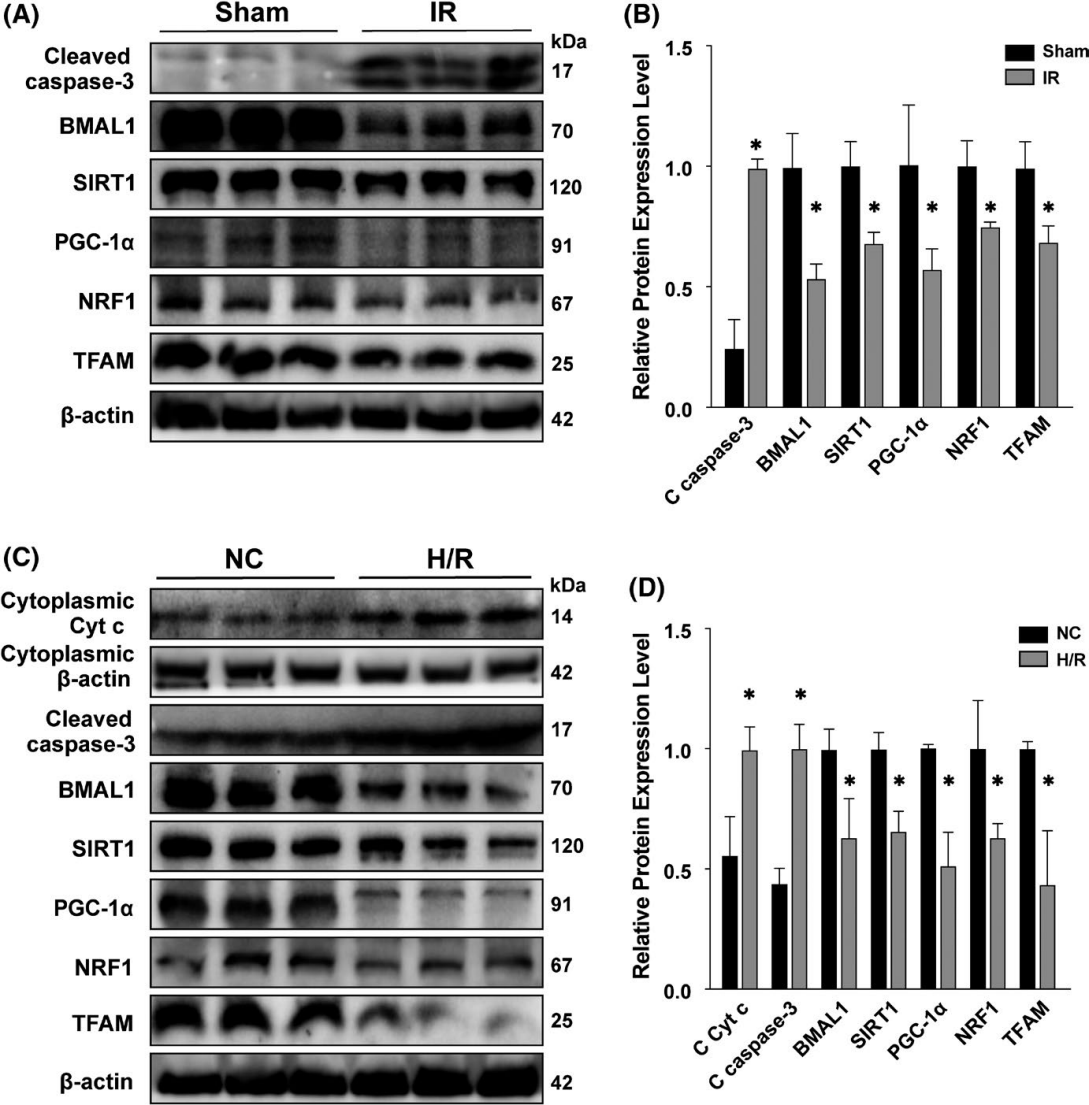
IR and H/R increased the expression of apoptosis‐related proteins and decreased the expression of BMAL1 and mitochondrial biogenesis‐related proteins in rat kidney and HK‐2 cells. (A) The protein expression levels of cleaved caspase‐3, BMAL1, SIRT1, PGC‐1α and mitochondrial biogenesis‐related proteins in the sham group and IR group. (B) Quantitative values of the relative expression levels of cleaved caspase‐3 BMAL1, SIRT1, PGC‐1α and mitochondrial biogenesis‐related proteins in the sham group and IR group. (C) The protein expression levels of cleaved caspase‐3, BMAL1, SIRT1, PGC‐1α and mitochondrial biogenesis‐related proteins in the NC group and H/R group. (D) Quantitative values of the relative expression levels of cleaved caspase‐3 BMAL1, SIRT1, PGC‐1α and mitochondrial biogenesis‐related proteins in the NC group and H/R group. Values are expressed as the mean ± SEM. ^*^ *p* < 0.05

The protein in rat kidneys was extracted and detected by Western blotting. Compared with that in the sham group, the protein level of the apoptosis‐related marker caspase‐3 was significantly increased in the IR group, while the expression of BMAL1, SIRT1, PGC‐1α and the mitochondrial biogenesis‐related proteins NRF1 and TFAM was significantly decreased (Figure 2A,B). In vitro, western blotting of cellular proteins showed the same results as the tissue protein analysis (Figure 2C,D). These results indicated that the expression of BMAL1 was decreased after IR and H/R, accompanied by downregulation of SIRT1/PGC‐1α and a decrease in mitochondrial biogenesis, which may exacerbate apoptosis.

### 3.3 Overexpression of BMAL1 can mitigate H/R injury by activating SIRT1/PGC‐1α to upregulate mitochondrial biogenesis levels

**FIGURE 3:**
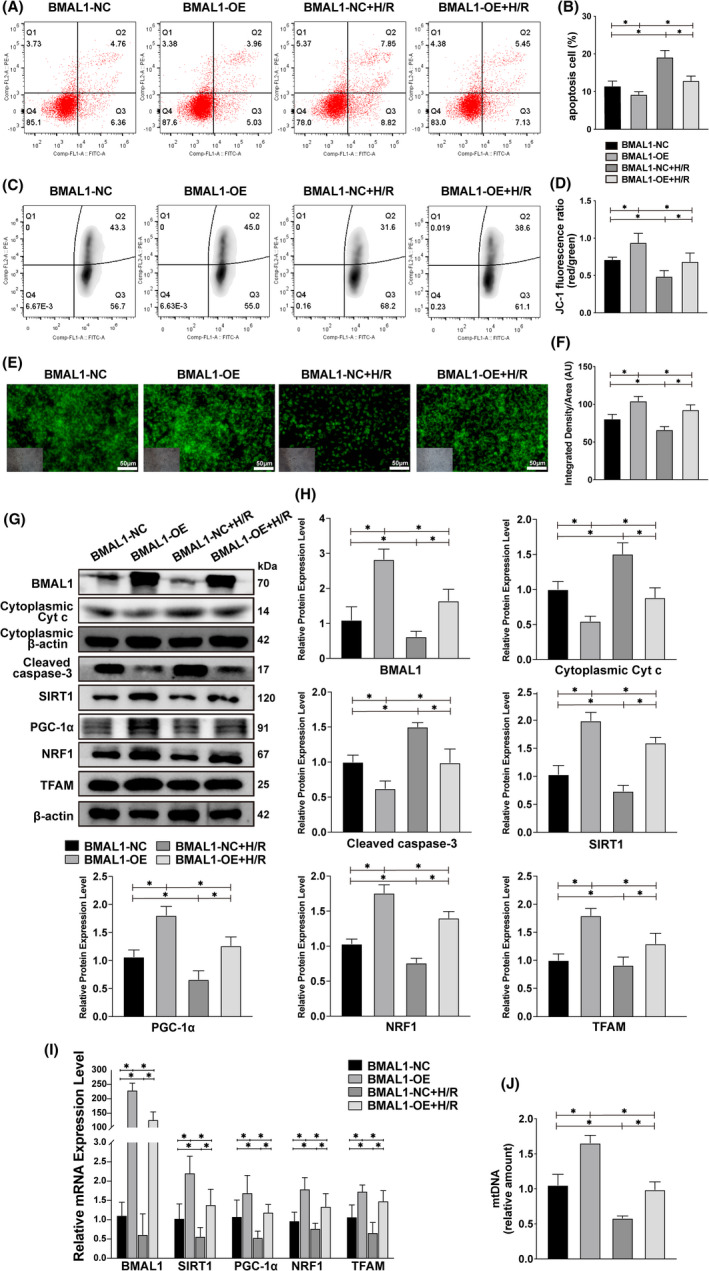
Overexpression of BMAL1 alleviates H/R‐induced apoptosis by mediating mitochondrial biogenesis. (A, B) Flow cytometry analysis of the effect of lentivirus infection or H/R treatment on apoptosis and the quantitative values. (C, D) Flow cytometry analysis of the effect of lentivirus infection or H/R treatment on MMP and the quantitative values. (E, F) Rhodamine 123 staining analysis of the effect of lentivirus infection or H/R treatment on MMP and the quantitative values. The lower left inset photographs show a bright field image under the same field of view. Scale bar = 50 µm. Magnification ×200. (G, H) Western blotting results for apoptosis‐related proteins and BMAL1, SIRT1, PGC‐1α, mitochondrial biogenesis‐related proteins and the quantitative values after lentivirus infection and H/R treatment. (I) Relative mRNA expression levels of BMAL1, SIRT1, PGC‐1α and mitochondrial biogenesis‐related proteins after lentivirus infection and H/R treatment. (J) Relative mtDNA amount after lentivirus infection and H/R treatment. Values are expressed as the mean ± SEM. ^*^ *p* < 0.05

To explore the role of BMAL1 in H/R‐induced renal injury and its relationship with SIRT1/PGC‐1α. BMAL1‐overexpressing HK‐2 cells were established using the pSLenti lentiviral vector system. In brief, HK‐2 cells were infected with overexpression lentivirus or control virus and cultured in an H/R or normal environment. Apoptosis and MMP were analysed via flow cytometry and fluorescence microscopy at the cellular level. Various markers were analysed via qRT‐PCR and Western blotting to detect mRNA and protein levels respectively. The flow cytometry results showed that the apoptosis rate in the BMAL1‐OE group was lower than that in the BMAL1‐NC group under both normal and H/R conditions. The overall apoptosis rate under H/R conditions was higher than that under normal conditions (Figure 3A,B). Similarly, the MMP results showed that the membrane potential under normal conditions was higher than that under H/R conditions (Figure 3C,D). Rhodamine 123 fluorescence showed that the fluorescence intensity of BMAL1‐OE group was significantly higher than that of BMAL1‐NC group, and H/R treatment could significantly reduce the fluorescence intensity, suggesting the decrease of MMP (Figure 3E,F). Overexpression of BMAL1 prevented a decrease in membrane potential. Furthermore, overexpression of BMAL1 decreased the expression of the apoptosis‐related proteins caspase‐3 and Cyt *c*, regardless of the culture environment (Figure 3G,H). Meanwhile, compared with the control group (BMAL1‐NC), the protein and mRNA levels of the mitochondrial biogenesis‐related markers NRF1, TFAM and SIRT1/PGC‐1α were significantly increased after BMAL1 overexpression (BMAL1‐OE) under both normal and H/R conditions (Figure 3G–I). Even after BMAL1 overexpression, the mRNA and protein expression levels of BMAL1, SIRT1, PGC‐1α, TFAM and NRF1 decreased due to the effect of H/R compared with levels in the BMAL1‐OE group under normal conditions (Figure 3G–I). Further analysis revealed that overexpression of BMAL1 increased mtDNA amount in HK‐2 cells under both normal and H/R conditions (Figure 3J), suggesting that BMAL1 may improve mitochondrial homeostasis by increasing the level of mitochondrial biogenesis. In conclusion, overexpression of BMAL1 can activate SIRT1/PGC‐1α and mediate mitochondrial biogenesis to reduce mitochondrial pathway apoptosis caused by H/R.

### 3.4 Knockdown of BMAL1 further exacerbated cell injury by mediating mitochondrial biogenesis under H/R conditions

**FIGURE 4:**
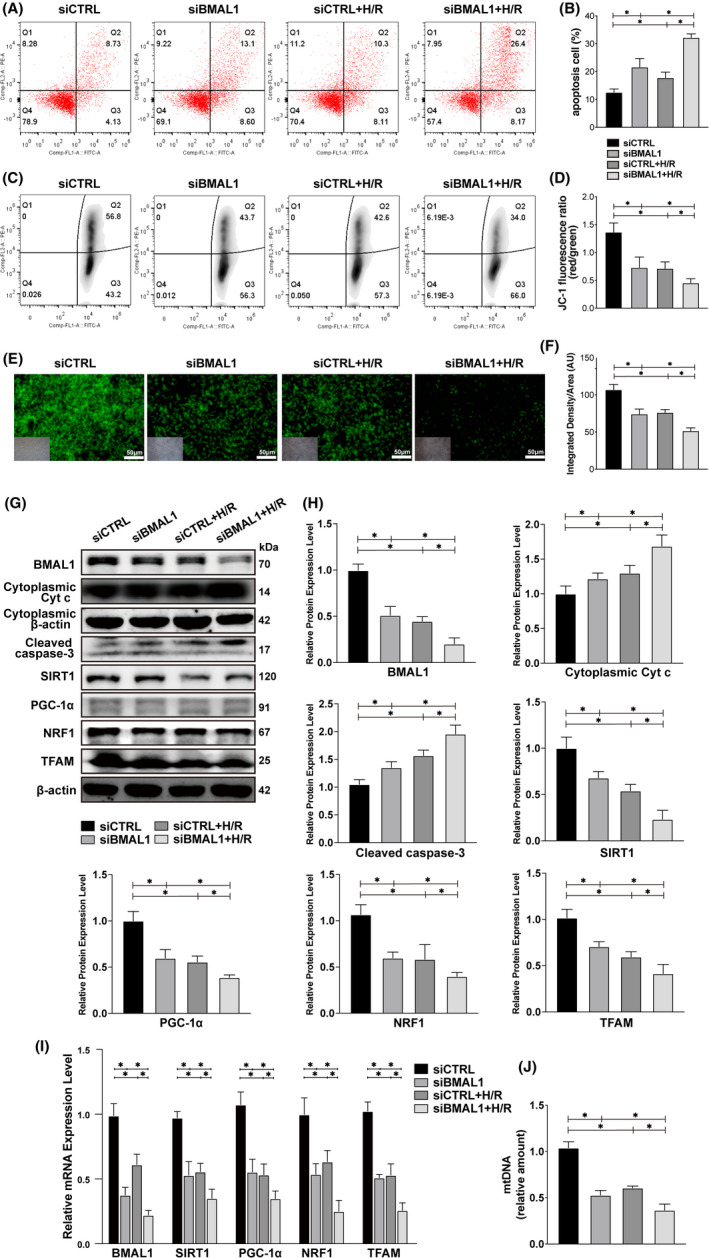
Knockdown of BMAL1 aggravates H/R‐induced apoptosis by mediating mitochondrial biogenesis. (A, B) Flow cytometry analysis of the effect of transfection with BMAL1 siRNA or H/R treatment on apoptosis and the quantitative values. (C, D) Flow cytometry analysis of the effect of transfection with BMAL1 siRNA or H/R treatment on MMP and the quantitative values. (E, F) Rhodamine 123 staining analysis of the effect of transfection with BMAL1 siRNA or H/R treatment on MMP and the quantitative values. The lower left inset photographs show a bright field image under the same field of view. Scale bar = 50 µm. Magnification ×200. (G, H) Western blotting results for apoptosis‐related proteins and BMAL1, SIRT1, PGC‐1α, mitochondrial biogenesis‐related proteins and the quantitative values after transfection with BMAL1 siRNA and H/R treatment. (I) Relative mRNA expression levels of BMAL1, SIRT1, PGC‐1α and mitochondrial biogenesis‐related proteins after transfection with BMAL1 siRNA and H/R treatment. (J) Relative mtDNA amount after transfection with BMAL1 siRNA and H/R treatment. Values are expressed as the mean ± SEM. ^*^ *p* < 0.05

To further assess the role of BMAL1 in human renal tubular cells, we knocked down BMAL1 expression in HK‐2 cells using an siRNA oligo system. After cells were transfected with either BMAL1 siRNA or control siRNA, we used flow cytometry to detect the changes in apoptosis and MMP at the cellular level. We found that the knockdown of BMAL1 led to an increase in apoptosis and a decrease in MMP, indicating that damage at the cellular level was aggravated and that the damage was further aggravated by culturing cells under H/R conditions (Figure 4A–D). Similarly, cell fluorescence showed a significant decrease in fluorescence intensity after BMAL1 was knocked down by siRNA, while H/R treatment caused a further decrease in fluorescence intensity, suggesting that the absence of BMAL11 and H/R treatment led to the loss of MMP (Figure 4E,F). Then, Western blotting and qRT–PCR were performed to detect the protein and mRNA levels of various markers. The Western blotting results showed that the protein expression levels of Cyt *c* and caspase‐3, which are related to apoptosis, increased to varying degrees after knockdown of BMAL1 expression or H/R (Figure 4G,H). The protein expression levels of SIRT1 and PGC‐1α and the mitochondrial biogenesis‐related markers TFAM and NRF1 were decreased after the knockdown of BMAL1 expression under normal conditions, and their expression levels were further reduced under H/R conditions (Figure 4G,H). The qRT–PCR results showed the same trend as the Western blotting results (Figure 4I). The mtDNA amount was significantly decreased after BMAL1 was knocked down as well as after H/R treatment (Figure 4J), indicated that both absence of BMAL1 and H/R can exacerbate apoptosis in the mitochondrial pathway by decreasing the level of mitochondrial biogenesis. The above results show that, regardless of the culture environment, deletion of BMAL1 leads to mitochondrial instability and apoptosis through SIRT1/PGC‐1α axis‐mediated mitochondrial biogenesis.

### 3.5 A SIRT1 inhibitor partially reversed the antiapoptotic effect of BMAL1 overexpression under H/R conditions by reducing SIRT1 activation

**FIGURE 5:**
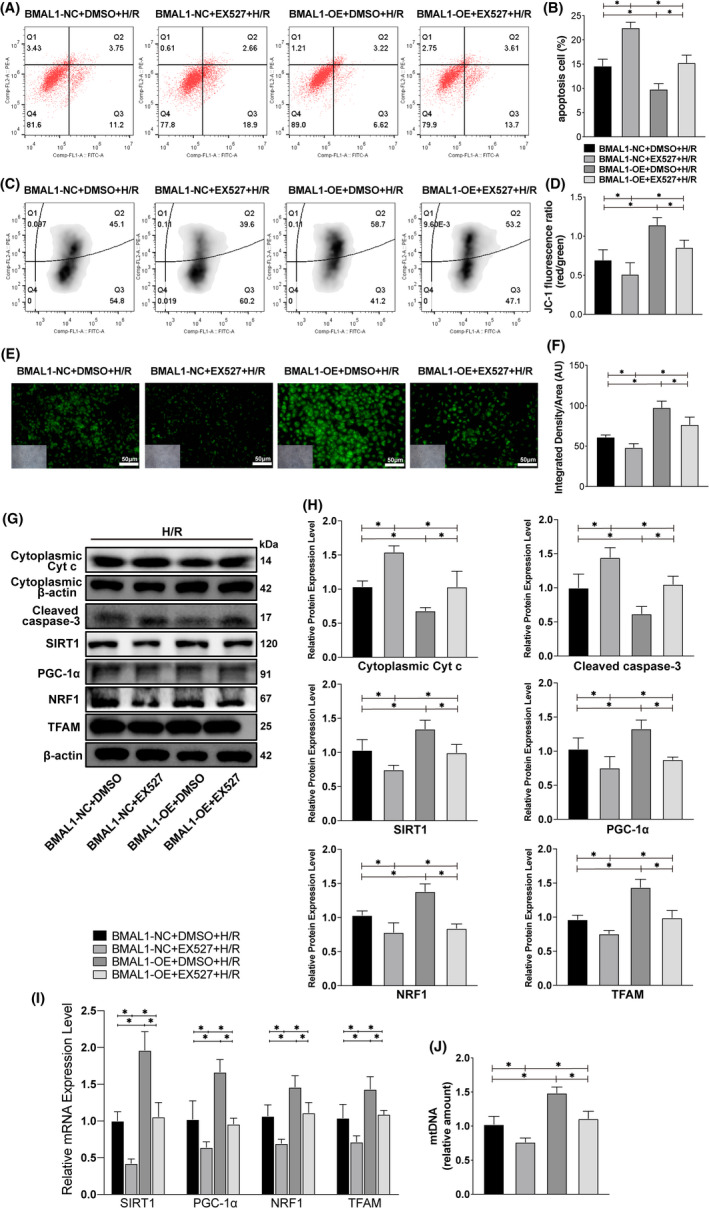
The SIRT1 inhibitor EX527 partially reversed the protective effect of BMAL1 overexpression by reducing SIRT1 activity under H/R conditions. (A, B) Flow cytometry analysis of the effect of lentivirus infection and EX527 application on apoptosis and the quantitative values. (C, D) Flow cytometry analysis of the effect of lentivirus infection and EX527 application on MMP and the quantitative values. (E, F) Rhodamine 123 staining analysis of the effect of lentivirus infection and EX527 application on MMP and the quantitative values. The lower left inset photographs show a bright field image under the same field of view. Scale bar = 50 µm. Magnification ×200. (G, H) Western blotting results for apoptosis‐related proteins, SIRT1, PGC‐1α, mitochondrial biogenesis‐related proteins and the quantitative values after lentivirus infection and EX527 application. (I) Relative mRNA expression levels of SIRT1, PGC‐1α and mitochondrial biogenesis‐related proteins after lentivirus infection and EX527 application. (J) Relative mtDNA amount after lentivirus infection and EX527 application. Values are expressed as the mean ± SEM. ^*^ *p* < 0.05

We treated HK‐2 cells with the SIRT1 inhibitor EX527 before H/R process to explore the mechanism and role of SIRT1 in the cell damage induced by H/R and the relationship between BMAL1, SIRT1 and mitochondrial biogenesis‐related markers. Flow cytometry results showed that the application of EX527 led to an increase in the apoptosis rate and a decrease in MMP (Figure 5A–D). The result of rhodamine 123 staining was identical to flow cytometric detection of MMP, overexpression of BMAL1 in cells significantly increased MMP, whereas the application of the SIRT1 inhibitor EX527 partially reversed the effect of overexpression, leading to a decrease in MMP (Figure 5E,F). As shown in Figure 5G,H, the expression levels of Cyt *c* and caspase‐3 decreased significantly and the expression levels of SIRT1, PGC‐1α, TFAM and NRF1 increased significantly after overexpression of BMAL1. The SIRT1 inhibitor EX527 significantly decreased SIRT1 activation and PGC‐1α, TFAM and NRF1 expression and increased Cyt *c* and caspase‐3 expression compared with the control groups (BMAL1‐NC + DMSO + H/R, BMAL1‐OE + DMSO + H/R). Similarly, qRT–PCR showed that the application of EX527 significantly reduced the mRNA expression of SIRT1, PGC‐1α, TFAM and NRF1 (Figure 5I). Moreover, mtDNA analysis revealed that the application of the SIRT1 inhibitor EX527 decreased mtDNA amount, indicating that EX527 reversed the promoting effect of BMAL1 overexpression on mitochondrial biogenesis and aggravated mitochondrial damage (Figure 5J). The above results indicated that the SIRT1 inhibitor EX527 could partially reverse the protective effect of BMAL1 overexpression on mitochondrial homeostasis and apoptosis in cells by inhibiting SIRT1 activity.

### 3.6 The SIRT1 agonist resveratrol partially reversed the aggravation of cell injury caused by knockdown of BMAL1 by increasing SIRT1 activity

**FIGURE 6:**
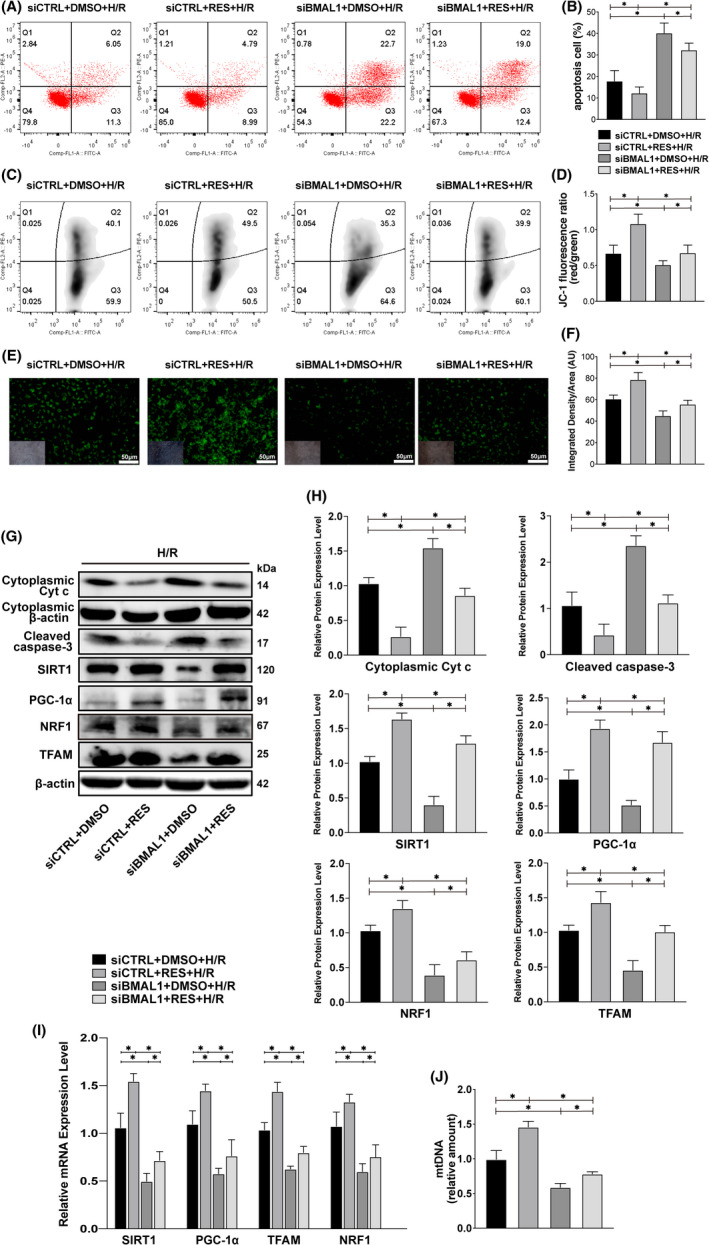
The SIRT1 agonist resveratrol partially reversed the cell damage caused by BMAL1 knockdown by reducing SIRT1 activity under H/R conditions. (A, B) Flow cytometry analysis of the effect of transfection with BMAL1 siRNA and resveratrol application on apoptosis and the quantitative values. (C, D) Flow cytometry analysis of the effect of transfection with BMAL1 siRNA and resveratrol application on MMP and the quantitative values. (E, F) Rhodamine 123 staining analysis of the effect of transfection with BMAL1 siRNA and resveratrol application on MMP and the quantitative values. The lower left inset photographs show a bright field image under the same field of view. Scale bar = 50 µm. Magnification ×200. (G, H) Western blotting results for apoptosis‐related proteins, SIRT1, PGC‐1α, mitochondrial biogenesis‐related proteins and the quantitative values after transfection with BMAL1 siRNA and resveratrol application. (I) Relative mRNA expression levels of SIRT1, PGC‐1α and mitochondrial biogenesis‐related proteins after transfection with BMAL1 siRNA and resveratrol application. (J) Relative mtDNA amount after transfection with BMAL1 siRNA and resveratrol application. Values are expressed as the mean ± SEM. ^*^ *p* < 0.05

Similarly, we stimulated BMAL1 knockdown HK‐2 cells with the SIRT1 agonist resveratrol before H/R process. We found that resveratrol reversed the increase in the apoptosis rate and the decrease in MMP caused by knockdown of BMAL1 (Figure 6A–F), accompanied by attenuation of the protein expression levels of caspase‐3 and Cyt *c* (Figure 6G,H). Moreover, due to the activation of SIRT1, the protein and mRNA expression levels of SIRT1, PGC‐1α, TFAM and NRF1 increased significantly compared with levels in the control group (Figure 6G–I). Furthermore, mtDNA analysis showed that the application of the SIRT1 agonist resveratrol significantly elevated the low level of mitochondrial biogenesis caused by BMAL1 knockdown and rescued mitochondrial homeostasis (Figure 6J). Given these findings, we presumed that BMAL1 regulates mitochondrial homeostasis and mitochondrial pathway apoptosis in HK‐2 cells in a SIRT1‐dependent manner.

### 3.7 The SIRT1 agonist resveratrol increased SIRT1 activity and attenuated mitochondrial apoptosis induced by IR

**FIGURE 7:**
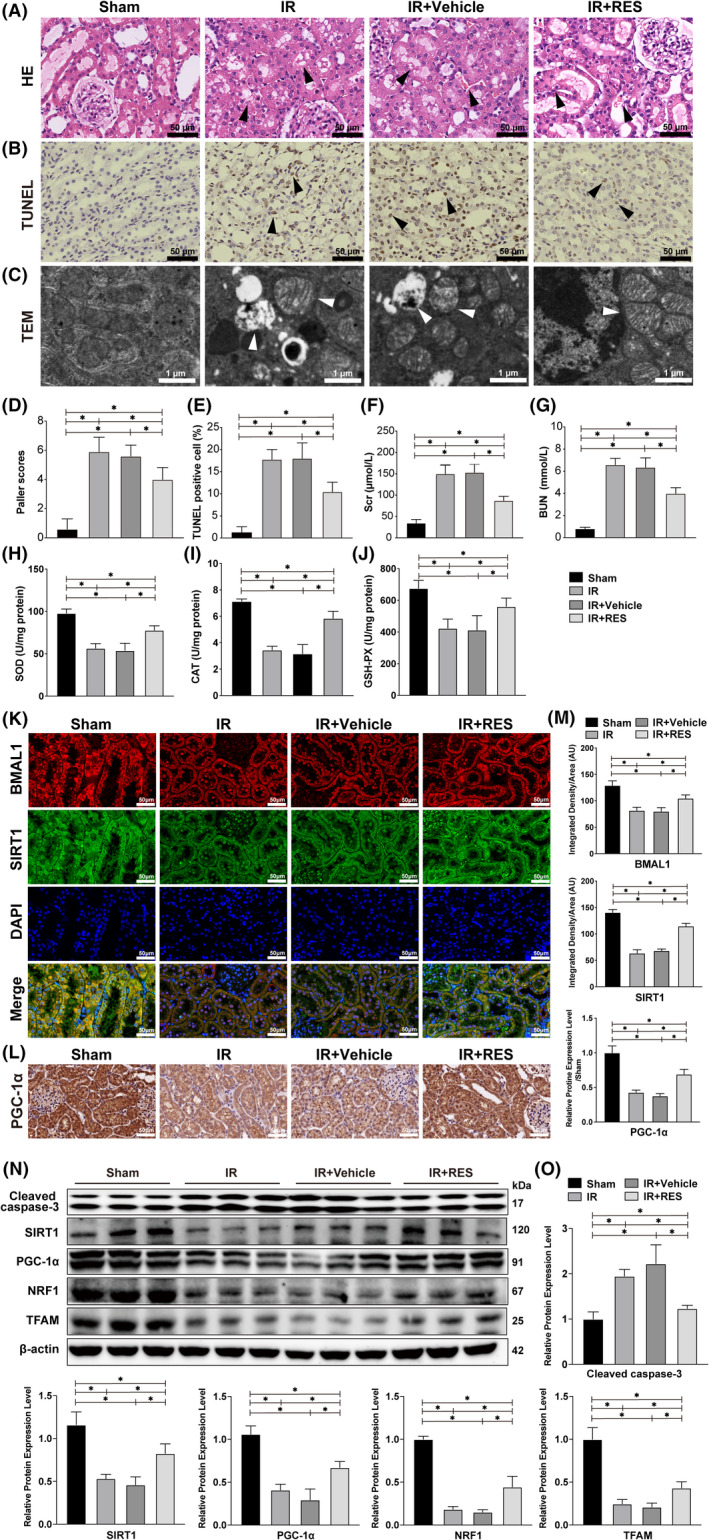
Resveratrol attenuated IR‐induced renal injury by increased SIRT1 and PGC‐1α activity. (A) HE staining of rat kidneys after the IR procedure and resveratrol application. The arrows in the figure indicate pathological damage (loss of brush border, haemorrhage, necrosis of renal tubular epithelial cells and inflammatory cell infiltration). Scale bar = 50 µm. Magnification ×400. (B) TUNEL staining of rat kidneys after the IR procedure and resveratrol application. The arrows in the figure indicate examples of TUNEL‐positive cells. Scale bar = 50 µm. Magnification ×400. (C) The substructural changes of rat kidney were detected by TEM. The arrows indicate examples of swollen and vacuolar mitochondria. Scale bar = 1 µm. Magnification ×5000. (D) Paller scores of each group after HE staining. (E) The proportion of TUNEL‐positive cells in each group after TUNEL staining. (F) The content of Scr in each group. (G) The content of BUN in each group. (H) The activity of SOD in each group. (I) The activity of CAT in each group. (J) The activity of GSH‐PX in each group. (K) Immunofluorescence staining of BMAL1 and SIRT1 in rat kidneys after IR and resveratrol application. Scale bar = 50 µm. Magnification ×400. (L) Immunohistochemical staining of PGC‐1α in rat kidneys after the IR procedure and resveratrol application. Scale bar = 50 µm. Magnification ×400. (M) Relative expression levels of BMAL1, SIRT1 and PGC‐1α. (N) Western blotting results for cleaved caspase‐3, SIRT1, PGC‐1α and mitochondrial biogenesis‐related proteins after IR and resveratrol application. (O) Quantitative values of the relative protein expression levels of cleaved caspase‐3, SIRT1, PGC‐1α, NRF1 and TFAM. Values are expressed as the mean ± SEM. ^*^ *p* < 0.05

We have shown that the protein expression levels of BMAL1 and SIRT1 in the kidneys of rats decreased significantly after IR and that BMAL1 regulates the mitochondrial pathway apoptosis of HK‐2 cells in a SIRT1‐dependent manner. To explore whether resveratrol has the same effect in vivo, rats in the experimental group were treated with intragastric administration of resveratrol for 14 days before IR. HE and TUNEL staining of sections revealed significant pathological changes in the kidney after IR, and the Paller scores and apoptosis rate increased significantly. The Paller score and apoptosis rate were alleviated after application of the agonist (Figure 7A,B,D,E). TEM results showed that the mitochondria in the kidney were abnormal in appearance after IR, and more swollen and vacuolated mitochondria were observed compared with the sham group. In the IR + RES group, no vacuolated mitochondria were observed, although swollen mitochondria were still present (Figure 7C). Meanwhile, the Scr and BUN levels were reduced significantly after the application of resveratrol (Figure 7F,G), the activities of SOD, CAT and GSH‐PX were also markedly increased after application of the agonists (Figure 7H–J). Immunofluorescence results also showed that IR decreased the expression of BMAL1 and SIRT1, while the expression of SIRT1 increased after the application of resveratrol (Figure 7K,M). Immunohistochemical results showed that the protein expression of PGC‐1α in renal tissue decreased significantly after IR, while the application of resveratrol increased PGC‐1α expression (Figure 7L,M). As shown in Figure 7N, the expression level of SIRT1 decreased markedly after IR treatment. As expected, the application of resveratrol alleviated the negative effect of IR on SIRT1 activity in the kidney. Moreover, the IR‐induced mitochondrial biogenic dysfunction, including decreased PGC‐1α, TFAM and NRF1 expression and increased caspase‐3 expression, was also reversed by resveratrol treatment (Figure 7N,O). Taken together, these data demonstrate that resveratrol can alleviate the mitochondrial instability and mitochondrial pathway apoptosis caused by IR by increasing SIRT1 activity.

## 4 DISCUSSION

**FIGURE 8:**
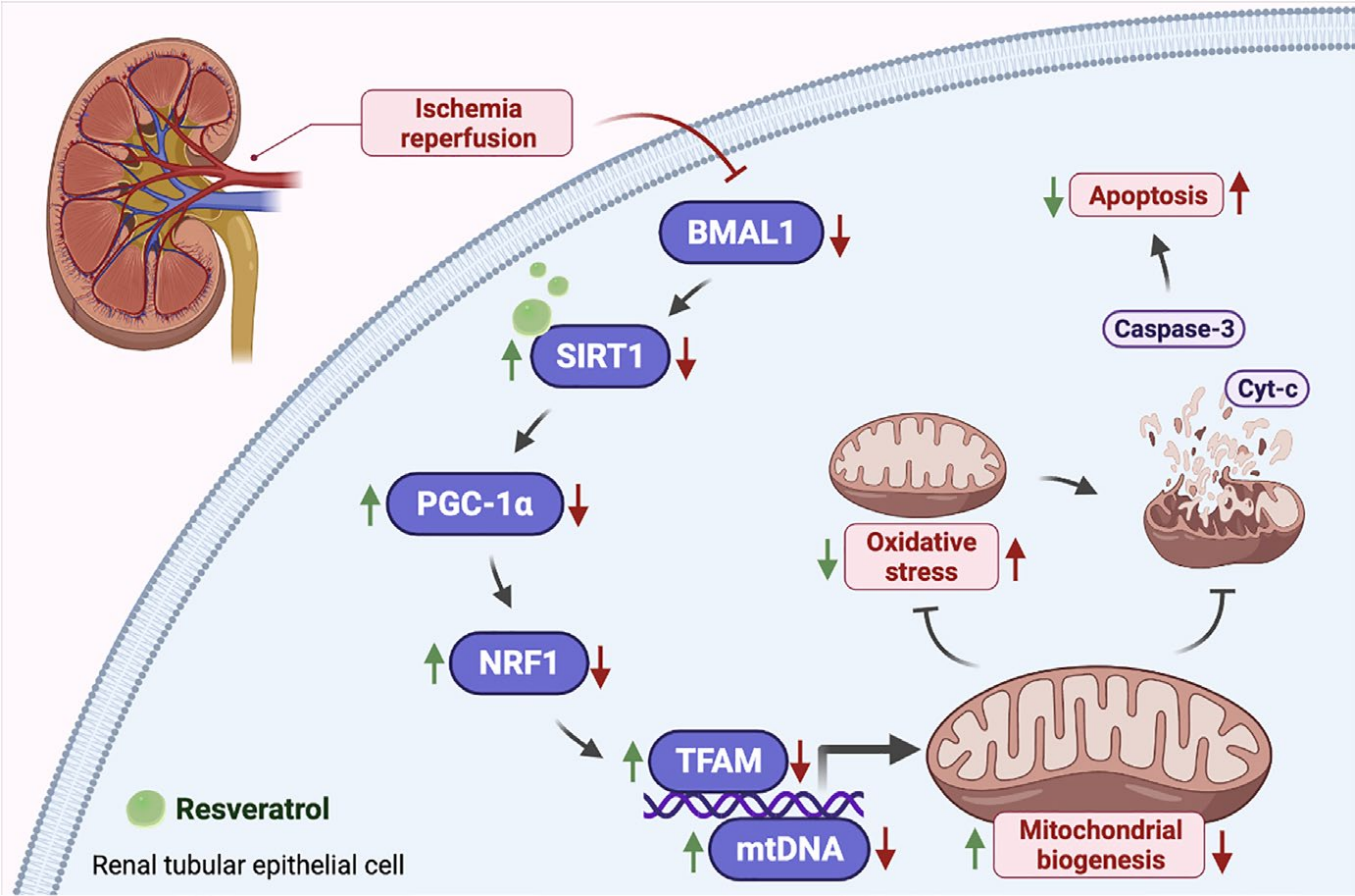
Schematic diagram of the involvement of BMAL1 in mitochondrial homeostasis during renal IRI. During renal IRI, the expression of BMAL1 is significantly downregulated, which reduces the activity of SIRT1. The decrement of SIRT1 activity downregulated the expression of PGC‐1α, which decreases the concentration of NRF1 and TFAM and inhibits the stability of mtDNA. The mitochondrial biogenesis level is inhibited by a decrease in mtDNA, thereby decreasing the mitochondrial homeostasis and increasing oxidative stress. An impaired mitochondrial homeostasis promotes mitochondrial outer membrane permeabilization (MOMP) and leads to the release of Cyt *c* and activation of the caspase cascade, which eventually leads to apoptosis (The figure was created in BioRender.com.)

Renal IRI is very common clinically, but due to its complicated pathogenesis, there is no effective treatment apart from supportive treatment.^4^ As a result of ischaemia, primary acute energy deficiency occurs, followed by secondary oxidative stress. During the reperfusion phase, both inflammatory response and apoptosis lead to tissue damage, with apoptosis as the dominant cause.^24^ With the deepened understanding of substructure, studies have identified that mitochondrial dynamics and mitochondrial biogenesis are the key processes to maintain mitochondrial homeostasis, and that impaired mitochondrial homeostasis may lead to renal tubular epithelial cell injury in IRI. The regulation of mitochondrial quality control is a promising therapeutic method for the prevention and treatment of AKI.^25^, ^26^, ^27^ Therefore, we sought to reveal how BMAL1 regulates mitochondrial homeostasis through in vivo and in vitro experiments. Our present results indicate that BMAL1 is involved in mitochondrial biogenesis and mitochondria‐mediated apoptosis in IRI, and we provide evidence that BMAL1 participates in the regulation of PGC‐1α, a key regulator of mitochondrial biogenesis, by regulating SIRT1 (Figure 8).

BMAL1 is the main transcriptional activator of the circadian clock, which has been shown to regulate the progress of various renal diseases.^7^, ^8^, ^9^, ^10^ However, researches on its function in renal IRI are limited. Previous studies have revealed that the expression level of BMAL1 in cardiac and cerebral tissues decreased significantly after IR,^17^, ^28^, ^29^ which is in line with our study showing that renal IR causes a dramatic decrease in BMAL1 expression level in renal tissues. In addition, many studies have pointed out that renal IRI can lead to a decrease in SIRT1 activity via multiple signalling pathways.^30^, ^31^, ^32^, ^33^ Previous studies have also explored the regulatory relationship between the circadian gene BMAL1 and SIRT1. Nakahata et al. reported that SIRT1 activity might be disrupted by the circadian rhythm of CLOCK:BMAL1 recruitment, through the rate‐limiting enzyme that controls the synthesis of the SIRT1 substrate NAD^+^ in mouse embryo fibroblasts.^34^ Research by YANG et al. has shown that the expression level of SIRT1 decreased with the downregulation of BMAL1, thus confirmed that a direct interaction of endogenous BMAL1 and SIRT1 protein in human cartilage.^18^ Furthermore, Zhou B et al. has found that BMAL1 binds to E‐box elements in the SIRT1 promoter and upregulates its transcription in liver and skeletal muscle.^19^, ^20^ In the present study, we found that the expression of SIRT1 is regulated by BMAL1 in renal tissues. Using lentivirus and siRNA in vitro, we regulated the expression level of BMAL1 intracellularly and found that the activity of SIRT1 was positively correlated with the expression of BMAL1. In conclusion, we have reached the same conclusions as previous studies that investigated the regulation of SIRT1 by BMAL1 in different tissues. In addition, we have confirmed that SIRT1 is regulated by BMAL1 in HK‐2 cells and rat kidney.

SIRT1‐mediated mitochondrial biogenesis plays an important role in disease progression.^35^ Studies have shown that drugs such as resveratrol, metformin and tetramethylpyrazine all target SIRT1 to increase mitochondrial biogenesis through PGC‐1α‐dependent pathways.^36^, ^37^, ^38^ Further investigations by Ham PB and Price NL have revealed the essential role of resveratrol in mediating AMPK to stimulate SITR1 and induce mitochondrial biogenesis. Resveratrol could increase the activities of cAMP, AMPK and NAD^+^. Enhanced NAD^+^ increased the activity of SIRT1 and deacetylation of PGC‐1α, and enhanced AMPK promoted phosphorylation of PGC‐1α. Phosphorylation and deacetylation of PGC‐1α are the necessary and sufficient condition for mitochondrial biogenesis.^39^, ^40^ We have found that IR results in a decrease in SIRT1 activity, which in turn leads to a decrease in PGC‐1α‐dependent mitochondrial biogenesis. After increasing SIRT1 activity using an exogenous SIRT1 agonist, the level of mitochondrial biogenesis induced by the PGC‐1α‐dependent pathway rebounds, and eventually the tissue/cell damage has been reduced. When exogenous SIRT1 inhibitor is used, the opposite effect is observed. Therefore, we speculated that in the renal IR model, BMAL1 regulates SIRT1 expression by binding to its transcriptional promoter and indirectly mediates PGC‐1α expression to regulate mitochondrial biogenesis.

Mitochondrial biogenesis is a process in which an organism generates new functioning mitochondria to enhance mitochondrial abundance and ATP production in response to the body's energy demands.^27^ It is a complex process strictly regulated by the nuclear genome (which encodes most mitochondrial proteins) and mitochondrial genome.^41^, ^42^ As a key regulator of mitochondrial biogenesis, PGC‐1α upregulates TFAM transcription by activating NRF1 activity, thereby promoting mitochondrial protein synthesis, mtDNA replication and functional mitochondrial regeneration.^43^, ^44^ Intracellular mtDNA abundance is directly regulated by TFAM, thus TFAM is an important factor in maintaining mitochondrial homeostasis.^45^ TFAM and mtDNA stabilize each other by binding to each other. Under pathological conditions, reduced TFAM level leads to mtDNA instability and degradation, resulting in impaired mitochondrial homeostasis and deficient renal function.^46^, ^47^ PGC‐1α also regulates the expression of mitochondrial antioxidant defence in cells. It is involved in the management of antioxidant enzymes and increases the activities of SOD, CAT and GSH‐PX to protect cells from mitochondrial dysfunction.^48^, ^49^ Further investigation by Olmos et al.^50^ revealed SIRT1 deacetylates PGC‐1α and FoxO3a in response to oxidative stress and regulates antioxidant gene. When the body is in a damaged state, such as oxidative stress increased and mitochondrial biogenesis, could save mitochondria from apoptosis.^51^, ^52^ When mitochondrial homeostasis is impaired, mitochondrial pathway apoptosis is elicited because of mitochondrial outer membrane permeabilization. As a result, proapoptotic factors such as Cyt *c* outflow, and the caspase cascade is activated, with caspase‐3 killing cells as an executor.^53^ Consistent with these reports, we found that the absence of PGC‐1α and TFAM decreased the activities of antioxidant enzymes and the amount of mtDNA, resulting in decrement of MMP and increment of mitochondrial pathway apoptosis eventually. Furthermore, the expression levels of NRF1 and TFAM changed with the expression level of PGC‐1α regulated by BMAL1, which confirms that BMAL1 regulates the repair of mitochondrial homeostasis.

In summary, our findings have demonstrated that BMAL1 regulates mitochondrial homeostasis through the SIRT1/PGC‐1α axis in renal IRI and identified a potential therapeutic target, SIRT1. In future studies, we will further explore the role of BMAL1 in renal IRI. Combing periodic rhythm oscillation of circadian genes, we will use adenovirus to interfere with the expression of target genes in experimental animals, or use tissue‐specific target gene deletion/overexpression animals to explore the further mechanism. Such studies may provide new insights into the role of circadian rhythm genes in the complex pathophysiology of renal IRI.

## CONFLICT OF INTEREST

The authors declare that they have no conflict of interest.

## AUTHOR CONTRIBUTION

**Peng Ye:** Conceptualization (equal); Methodology (equal); Writing – original draft (equal). **Wei Li:** Methodology (equal); Writing – review & editing (equal). **Xin Huang:** Methodology (equal). **Sheng Zhao:** Software (equal). **Wu Chen:** Formal analysis (equal). **Yuqi Xia:** Formal analysis (equal). **Weimin Yu:** Investigation (equal). **Ting Rao:** Data curation (equal). **Jin‐Zhuo Ning:** Data curation (equal). **Xiangjun Zhou:** Investigation (equal). **Yuan Ruan:** Project administration (equal). **Fan Cheng:** Funding acquisition (equal); Project administration (equal).

## Supporting information

Supplementary Material

## Data Availability

The data that support the findings of this study are available from the corresponding author upon reasonable request.
