# Supplementary material for: BMAL1 regulates mitochondrial homeostasis in renal ischaemia‐reperfusion injury by mediating the SIRT1/PGC‐1α axis
Source: J Cell Mol Med. 2022 Feb 17;26(7):1994–2009. doi: 10.1111/jcmm.17223 (PMC8980910; doi:10.1111/jcmm.17223)
Supplement: Supplementary file 1 — Supplementary Material [file JCMM-26-1994-s001.docx]

**Supplemental Experimental Procedures**

**Lentivirus infection and siRNA transfection**

The lentivirus and siRNA were purchased from OBiO Technology (Shanghai). The lentiviral expression vector was used for BMAL1 gene (NM_001297719.2) delivery and stable overexpression and was designated BMAL1-OE (pSLenti-EF1-P2A-Puro-CMV-ARNTL-3xFlag-WPRE). The empty vector was used as a negative control (BMAL1-NC; pSLenti-EF1-P2A-Puro-CMV-MCS-3xFlag-WPRE). The MOI was 20. Puromycin (50 µg/ml) was used to screen uninfected cells, and the surviving cells were further cultured and expanded. The siRNA sequence used to knock down BMAL1 (siBMAL1) was forward: 5'-CCGAAUGCUGAGGAATT-3' and reverse: 5'-UUCCUCAGCAAUCAUUCGGTT-3'. The sequence of the negative control was forward: 5'-UUCUCCGAACGUGUCACGUTT-3' and reverse: 5'-ACGUGACACGUUCGGAGAATT-3'. HK-2 cells were transfected with siBMAL1 and siCTRL using Lipofectamine 2000 (11668027, Thermo Fisher) according to the manufacturer's protocol.

**Antioxidant enzyme activity measurement**

Kidney tissues were homogenized after being mixed with cold saline in a 1:9 ratio. The homogenate was centrifuged at 3000 rpm at 4°C for 10 minutes and the supernatant was taken for subsequent antioxidant enzyme activity detection. Superoxide dismutase (SOD) activity, catalase (CAT) activity and glutathione peroxidase (GSH-PX) activity in kidney tissues were detected with commercial kits respectively (A001, A007, A005, Nanjing Jiancheng Bioengineering Institute) according to the manufacturer's instructions and results were obtianed with a microplate absorbance reader.

**Immunohistochemical staining**

After paraffin sections were dewaxed and hydrated with xylene and alcohol, the tissues were blocked with 0.3% hydrogen peroxide in methanol for 15 minutes and with 5% goat serum in PBS for 30 minutes at room temperature. Then, the tissue sections were incubated with primary antibody (anti-PGC-1α, 1:200, NBP1-04676, NOVUS) at 4°C overnight. Next, the sections were incubated with biotinylated anti-rabbit secondary antibodies (A0279, Beyotime) and reagents from a SABC-HRP Kit (P0603, Beyotime) at room temperature for 1 hour and then visualized using a DAB Horseradish Peroxidase Color Development Kit (P0202, Beyotime). Afterward, the sections were counterstained with 5% hematoxylin and observed.

**Immunofluorescence staining**

After paraffin sections were dewaxed and hydrated with xylene and alcohol, antigen repair was performed with citrate antigen retrieval solution (P0081, Beyotime). The samples were then blocked at room temperature for 1 hour using immunol standing blocking buffer (P0102, Beyotime). Subsequently, the samples were incubated with primary antibodies against BMAL1 (1:100, NB100-2288S, NOVUS) and SIRT1 (1:100, 8469S, CST) at 4°C overnight. Afterward, the samples were incubated with the appropriate fluorescein-labeled IgG secondary antibody for 1 hour and then stained with 5 µg/ml DAPI for 5 minutes.

**Apoptosis assay**

Treated HK-2 cells were digested with trypsin without EDTA, washed with cold PBS and resuspended in buffer at a density of 5×10^6^ cells/mL. Then, according to the manufacturer's instructions, the cells were double stained with fluorescein isothiocyanate (FITC) and propidium iodide (PI) using an FITC Annexin V Apoptosis Detection Kit I (556547, BD Pharmingen) Subsequently, the stained cells were detected by flow cytometry (CytoFLEX, BECKMAN COULTER).

**Mitochondrial membrane potential (MMP) assay**

JC-1 staining and rhodamine 123 staining were used to detect changes in MMP. JC-1 staining was used for flow cytometry, whereas rhodamine 123 was used for fluorescence microscopy observation. For flow cytometry, treated HK-2 cells were digested with trypsin without EDTA, washed with cold PBS and resuspended in buffer at a density of 5×10^6^ cells/mL, then the cells were stained with JC-1 dye using a Mitochondrial Membrane Potential Assay Kit with JC-1 (C2006, Beyotime) and detected by flow cytometer (CytoFLEX, BECKMAN COULTER). For fluorescence microscopy observation, after the cells in 6 well plates were washed twice with PBS, 1 ml of rhodamine 123 staining solution (C2008s, Beyotime) was added per well and incubated at 37°C for 1 hour. After staining was completed, cells were observed using a fluorescence microscope (IX51, Olympus).

**Gene expression and mtDNA analysis**

TRIzol (R0016, Beyotime) was used to extract total RNA from treated cells. Subsequently, RNA was reverse transcribed into cDNA using HiScript® III RT SuperMix for qPCR (R323-01, Vazyme). Then, qRT–PCR was performed on a QuantStudio 6 system (Applied Biosystems, Thermo Fisher Scientific) using a HiScript® II One Step qRT–PCR SYBR Green Kit (Q221-01, Vazyme). The primer sequences used for qRT-PCR were as follows: BMAL1 forward, 5'- AAGGGAAGCTCACAGTCAGAT-3' and reverse, 5'-GGACATTGCGTTGCATGTTGG-3'; SIRT1 forward, 5'- TGTGTCATAGGTTAGGTGGTGA-3', and reverse, 5'- AGCCAATTCTTTTTGTGTTCGTG-3'; PGC-1α forward, 5'-TCTGAGTCTGTATGGAGTGACAT-3', and reverse, 5'- CCAAGTCGTTCACATCTAGTTCA-3'; TFAM forward, 5'- GTCACTGCCTCATCCACC-3', and reverse, 5'- CCGCCCTATAAGCATCTT-3'; NRF1 forward, 5'- AGGAACACGGAGTGACCCAA-3', and reverse, 5'- TATGCTCGGTGTAAGTAGCCA-3'; and actin forward, 5'- TCAAGAAAGGGTGTAACGCAACTA-3', and reverse, 5'- CGACAGGATGCAGAAGGAGAT-3'. β-actin was used as the housekeeping gene, and the 2− ^∆∆^Ct method was used to calculate the relative expression levels. For mtDNA analysis, total DNA was extracted from treated cells using Universal Genomic DNA Purification Mini Spin Kit (D0063, Beyotime) and 10 ng DNA was used for further qRT-PCR. The mitochondrial ND1 (MT-ND1 forward, 5'-CACTTTCCACACAGACATCA-3', and reverse, 5'-TGGTTAGGCTGGTGTTAGGG-3') was used to amplify the mtDNA and nuclear beta-2 microglobulin (B2M forward, 5'-TGTTCCTGCTGGGTAGCTCT-3' and reverse, 5'-CCTCCATGATGCTGCTTACA-3') was used to normalize the mtDNA amount.
